# Intraspecific Variation in Physiological Condition of Reef-Building Corals Associated with Differential Levels of Chronic Disturbance

**DOI:** 10.1371/journal.pone.0091529

**Published:** 2014-03-13

**Authors:** Chiara Pisapia, Kristen Anderson, Morgan S. Pratchett

**Affiliations:** 1 ARC Centre of Excellence for Coral Reef Studies, James Cook University, Townsville, Australia; 2 AIMS@JCU,Australian Institute of Marine Science, School of Marine Biology, James Cook University, Townsville, Australia; Institute of Oceanology, Chinese Academy of Sciences, China

## Abstract

Even in the absence of major disturbances (e.g., cyclones, bleaching), corals are subject to high levels of partial or whole-colony mortality, often caused by chronic and small-scale disturbances. Depending on levels of background mortality, these chronic disturbances may undermine individual fitness and have significant consequences on the ability of colonies to withstand subsequent acute disturbances or environmental change. This study quantified intraspecific variations in physiological condition (measured based on total lipid content and zooxanthellae density) through time in adult colonies of two common and widespread coral species (*Acropora spathulata* and *Pocillopora damicornis*), subject to different levels of biological and physical disturbances along the most disturbed reef habitat, the crest. Marked intraspecific variation in the physiological condition of *A. spathulata* was clearly linked to differences in local disturbance regimes and habitat. Specifically, zooxanthellae density decreased (r^2^ = 26, df = 5,42, p<0.02, B =  −121255, p = 0.03) and total lipid content increased (r^2^ = 14, df = 5,42, p = 0.01, B = 0.9, p = 0.01) with increasing distance from exposed crests. Moreover, zooxanthellae density was strongly and negatively correlated with the individual level of partial mortality (r^2^ = 26, df = 5,42, p<0.02, B =  −7386077, p = 0.01). Conversely, *P. damicornis* exhibited very limited intraspecific variation in physiological condition, despite marked differences in levels of partial mortality. This is the first study to relate intraspecific variation in the condition of corals to localized differences in chronic disturbance regimes. The next step is to ascertain whether these differences have further ramifications for susceptibility to periodic acute disturbances, such as climate-induced coral bleaching.

## Introduction

Coral reefs are very dynamic ecosystems, impacted by a variety of natural and anthropogenic processes, which may vary in scale, frequency, and intensity [1]. Even in the absence of major disturbances (e.g., cyclones, bleaching or outbreaks of crown-of-thorns starfish), corals are still subject to a range of chronic, often small-scale disturbances that cause relatively high rates of background mortality (annual background mortality rates can generally vary from 1 to 30%: [2]–[5]). These background mortality agents (such as predation, competition and disease) are a normal part of the natural dynamics and turnover in coral populations and communities [6]–[8]. However, increases in prevalence and impact of chronic disturbances undermine the resilience of coral colonies and populations [4], [5], [8], [9], [10], which are subject to ever-increasing threats from climate change and other more direct anthropogenic disturbances [11], [12].

Background mortality agents can trigger complex responses in corals that may affect colony physiological condition, alter demographic performance, especially growth [13]–[15] and reproduction [15], [16], [17] and they can therefore have significant consequences on the ability of colonies to withstand and survive periodic acute disturbances and environmental changes [18]. Intraspecific competition, for example, can substantially reduce fitness and growth rates of colonies engaged in competitive interactions [14]. Tanner 1997 documented a reduction in growth rates from 120 to 35% in *Acropora hyacinthus* when engaged in competitive interactions, and a decrease in growth from 45 to −16% in *Pocillopora damicornis*. Similarly, chronic predation can inflict a significant energetic cost to prey corals and may accelerate rates of coral decline following a disturbance [19]. Coral grazing fishes are a potentially important source of background coral mortality [19], even when they do not leave any visible signs of damage on coral colonies [20]. Rates of tissue removal from individual coral colonies can be considerable (16.75±0.30 bites per 20 min, [19]) and this chronic removal of live tissue can have potentially important consequences for colony fitness. Similarly, sedimentation can affect coral physiological condition by exerting significant energetic costs due to the removal of particles from colonies and limit energy availability due to reduced light and photosynthetic activity [15], [21]. *Siderastrea siderea* reduced linear extension rates from 3.5 mm to 3 mm three years following an oil spill, which caused increased sedimentation levels [21].

The physiological condition of a colony is largely determined by the energy available and by the partitioning of energy reserves among maintenance, growth, and reproduction [22]. Energy within a colony is a limited resource and it is distributed among costly life history processes. If a coral invests heavily in repairing tissues damaged by chronic predation or sedimentation, or is investing heavily in interspecific competition, then this will reduce resources available for growth and reproduction. Evidences of energy trade-offs have been widely documented in corals, with injury often causing a decline in growth [23], [24] or fecundity [25]. Moreover, diversion of essential energy reserves may undermine the capacity of corals to withstand periodic acute disturbances, such as anomalous temperatures that cause widespread bleaching [22]. When injured, corals often divert energy towards regeneration of lost tissue, and species with high regenerative capacity (such as *Acropora* spp) being able to fully heal the injury in less than 80 days [23]. However, environmental stresses, large lesions and competition may impair regeneration and hence compromise survival [23], [26]. The bare skeleton resulting from tissue loss can be colonized by algae, pathogens or bioeroders, which may undermine the integrity of the colony [27], [28]. These organisms may later compete with the coral for food and space, or cause structural damage to the coral skeleton [27], [28].

The capacity of corals to withstand ongoing disturbances is strongly size-dependent, with small colonies being more vulnerable to whole-colony mortality than larger ones [6]. Corals as modular organisms are made up of repeated units (polyps), each of which can function and survive as physiologically independent entities. However, partial mortality and the consequent decline in the total number of polyps that make up a colony can greatly reduce individual fitness and resilience [6], [17], [29]. A reduction in size results in fewer polyps available to support colony vital processes and will generally reduce survivorship [6], [30], growth [23], [24], reproduction and regeneration [15]. Large colonies have greater regenerative abilities [6], [8], growth [31], are more fecund [17] and have lower rates of total mortality compared to smaller colonies [6], [17]. Likelihood of survival in larger colonies is greater than smaller ones because there is a higher probability that part of the colony may remain unaffected [32]. Particularly, following a disturbance, big colonies can make a disproportionate contribution to population as they produce more eggs per unit area [17].

Intra-specific variation of corals in responses to stresses is largely due to genotypic and phenotypic variation among both corals and their zooxanthellae [33]–[35], however the disturbance history and current physiological condition of individual colonies may also play a critical role. The exhaustion of energy available to maintain vital processes represents a physiologically critical threshold for survival [36]. During a bleaching event for instance, a key determinant for survival and recovery of a coral is its amount of lipid reserves [22], as they can. When bleaching occurs the energy acquisition by the zooxanthellae stops, hence the coral must use its energy reserves accumulated in the form of lipids in order to survive [38]–[41]. So colonies in good physiological conditions, with a great magnitude of lipid reserves, are more likely to survive and recover from a bleaching event, than colonies with lower level of lipid reserves [22], [42]. Also colonies which survived a previous disturbance and are potentially in good physiological condition, can substantially contribute to community recover through their growth and through their reproductive output [17], [43], [44].

The purpose of this study is to quantify intra-specific variation in physiological condition (specifically, total lipid content and zooxanthellae density) through time in adult colonies exposed to several biological and environmental factors. Variation in colony condition among individuals may account for differences in susceptibility to disturbances. Many studies have documented significant variation in the capacity of corals to withstand and recover from major disturbances [4], [8], [45], [46], but the underlying basis of this variation is still poorly understood. Most of these studies have focused on among-species variability for stress resistance. Hoegh-Guldberg [47] suggested that in the aftermath of climate change some coral species are more likely to adapt and survive better than others. But still little is known on intraspecific variability to environmental changes.

## Methods

### Ethics Statement

The activities for this study were conducted under permission from the Great Barrier.

Reef Marine Park Authority (Permit Number G12/35017.1). Visual censuses of fishes and benthic communities were conducted during this study; one coral branch per colony was collected in May and one in October.

### Chronic Disturbances

This study was conducted at Lizard Island (14°40′S, 145°27′E) in the northern Great Barrier Reef, Australia. 24 colonies, ranging in size from 9 cm up to 35 cm diameter, of *Pocillopora damicornis* and *Acropora spathulata* were individually tagged and sampled in May and October 2012 to test for intraspecific differences in physiological condition. At the same time, detailed observations were undertaken to quantify intra-specific differences in background disturbance regimes (NB. There were no major bleaching events or other acute disturbances during the conduct of this study). Colonies at the same depth were selected from the reef crest in two different sites, one sheltered and one in the windward side of the island. For each coral colony we measured the distance from the reef crest (presumed to reflect colony physical position in respect of local hydrodynamic regime), proportional tissue loss attributable to coral competition and/or coral disease, and also rates of predation by corallivorous fishes. Variation in the level of predation among individual coral colonies was documented using GoPro cameras, deployed to record the total number of bites taken by all corallivorous fishes within replicate one-hour periods. The fish species and size were also recorded. Partial mortality was measured by quantifying the exact proportion of dead versus living tissue within the overall physical extent of each coral colony, using the software Image J. Growth rates were also calculated comparing colony surface area from pictures in May and in October for each individual colony.

### Colony Physiological Condition

Colony condition was assessed based on total lipid content and zooxanthellae density. The size of lipid reserves is a good measure of colony condition because it represents an alternative source of fixed carbon, which can be allocated to vital processes such as growth or reproduction. Lipid reserves can also allow the host to meet its daily metabolic energy needs in absence of endosymbionts, such as during a bleaching event [42]. Similarly, the symbiotic relationship between the coral colonies and the symbionts makes zooxanthellae density a good proxy of coral condition [48]. Zooxanthellae density has been shown to decrease in response to chronic stresses such as exposure to both low and high temperature [49], sedimentation [50], disease [51], and water quality [52], [53], and has been widely measured to assess coral condition in response to stimulants, as well as natural variation in environmental factors [52], [53], [54], [55]. To measure both total lipid content and zooxanthellae density, one branch was collected from each of the tagged colony in May and October. To minimize within-branch variability in lipids, only central inner branches were collected [56].

Branches were fixed in 10% formalin seawater and decalcified in 5% Formic acid for 1 day followed by 10% formic acid for 5 days and then stored in 70% ethanol. To extract total lipids, coral branches were dried in the oven at 55°C for 24 h, weighed and placed in a solution of chloroform: methanol (2∶1, v:v) to dissolve the lipids [57]. The tissues were redried at 55°C overnight and reweighed. The difference in weight was due to lipids loss, with total lipid content then expressed as percentage of dry weight. Total lipid content was analysed instead of lipid classes because of the total lipids, triacylglycerol and wax esters are the main storage lipids in corals, and can account for 40–73% of total lipids [58]–[60].

Zooxanthellae density (per unit surface area (cells/cm^2^)) was quantified for each coral based on samples (5 mm×5 mm) from the collected branch (4 replicates per branch). Each sample was homogenized and the ground solution was examined on a glass slide under a microscope and counts were normalized to coral surface area, following McCowan et al. [61].

Data were deposited on Figshare and are available at http://dx.doi.org/10.6084/m9.figshare.928440.

### Data Analyses

To test whether there were significant differences in partial mortality, in total lipid content, in zooxanthellae density, in competition and in the number of fish bites, between May and October, a series of paired t-tests were carried out for each variable. Proportional mortality of individual coral colonies was Arcsin transformed prior to analyses. A One-Way ANOVA was carried out to further investigate whether colonies exposed to predation had lower lipid content than colonies that did not receive any bite. To test whether physiological condition of coral colonies relates to biological and physical disturbance regimes, we used a stepwise Multiple Regression model, testing the extent to which i) partial mortality, 2) mean number of fish bites, 3) extent of coral competition, 4) colony size, 5) distance from crest, and 6) Site, explained intraspecific variation in either total lipid content or zooxanthelae density for each coral species. Separate analyses were carried out for total lipid content and zooxanthellae density. Bivariate correlations were also used to test for any relationship between zooxanthellae density and total lipid content in each coral species.

## Results

### Chronic Disturbances

**Figure 1:**
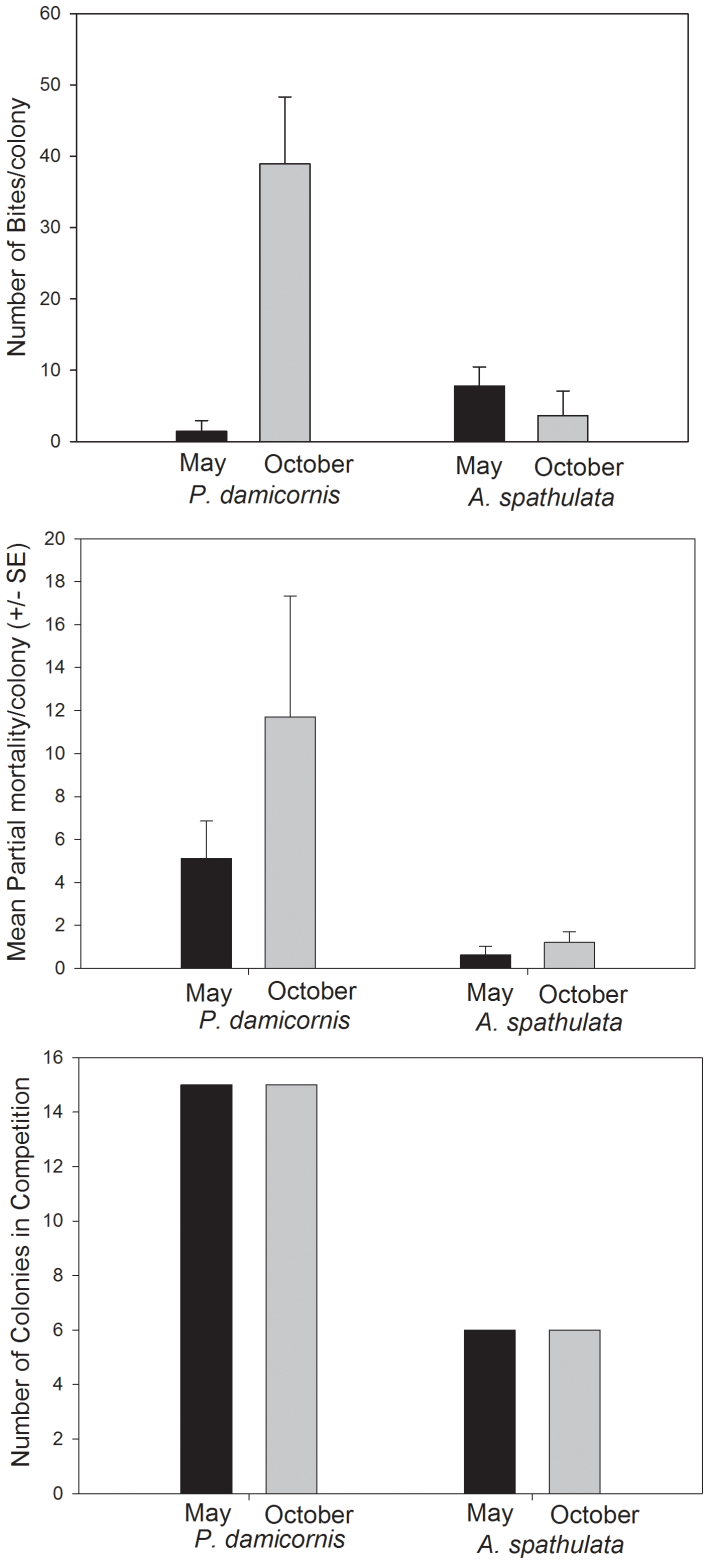
Chronic disturbance regimes in May and October in the two reef-building corals *P. damicornis* and *A. spathulata*. A) Predation – mean no. of bites taken per colony in replicate three-minute observations, where Go Pro cameras were used to record the total number of bites taken by all corallivorous fishes (mostly, butterflyfishes), B) Partial mortality –proportional of dead versus living tissue within the overall physical extent of each coral colony, C) – number of colonies engaged in competitive interactions.

Competitive interactions and partial mortality were constant between May and October in both coral species (Fig. 1). Only the number of fish bites differed significantly with time, being 23 times higher in October than in May for *P. damicornis* (paired t-test, p<0.05). Some colonies received few bites in May (from 0 to 4 bites per hour) while they were exposed to high predation pressure in October (163 and 392 bites per hour). In *A. spathulata*, overall predation pressure was two times higher in October, but given marked intra-specific variation this was not statistically significant (Fig. 1). Bite rate varied among colonies in *P. damicornis*, ranging from 0 to >100 bites per hour among colonies. In both coral species, the colonies that received most bites in May were not the same ones that received most bites in October, while some colonies did not receive any bite in either May or October.

In *P. damicornis* the majority of the colonies were smaller than 1000 cm^2^, with colony surface area ranging from 161 cm^2^ to 679 cm^2^, while in *A. spathulata* colony surface area ranged from 160 cm^2^ up to 1.830 cm^2^. Colony growth (expressed as changes in colony surface area) from May to October in *A. spathulata* was 118.3 cm^2^, while *P. damicornis* showed a negative growth rate (−10.3 cm^2^) due to partial mortality.

### Intraspecific Variation in Colony Condition

**Figure 2:**
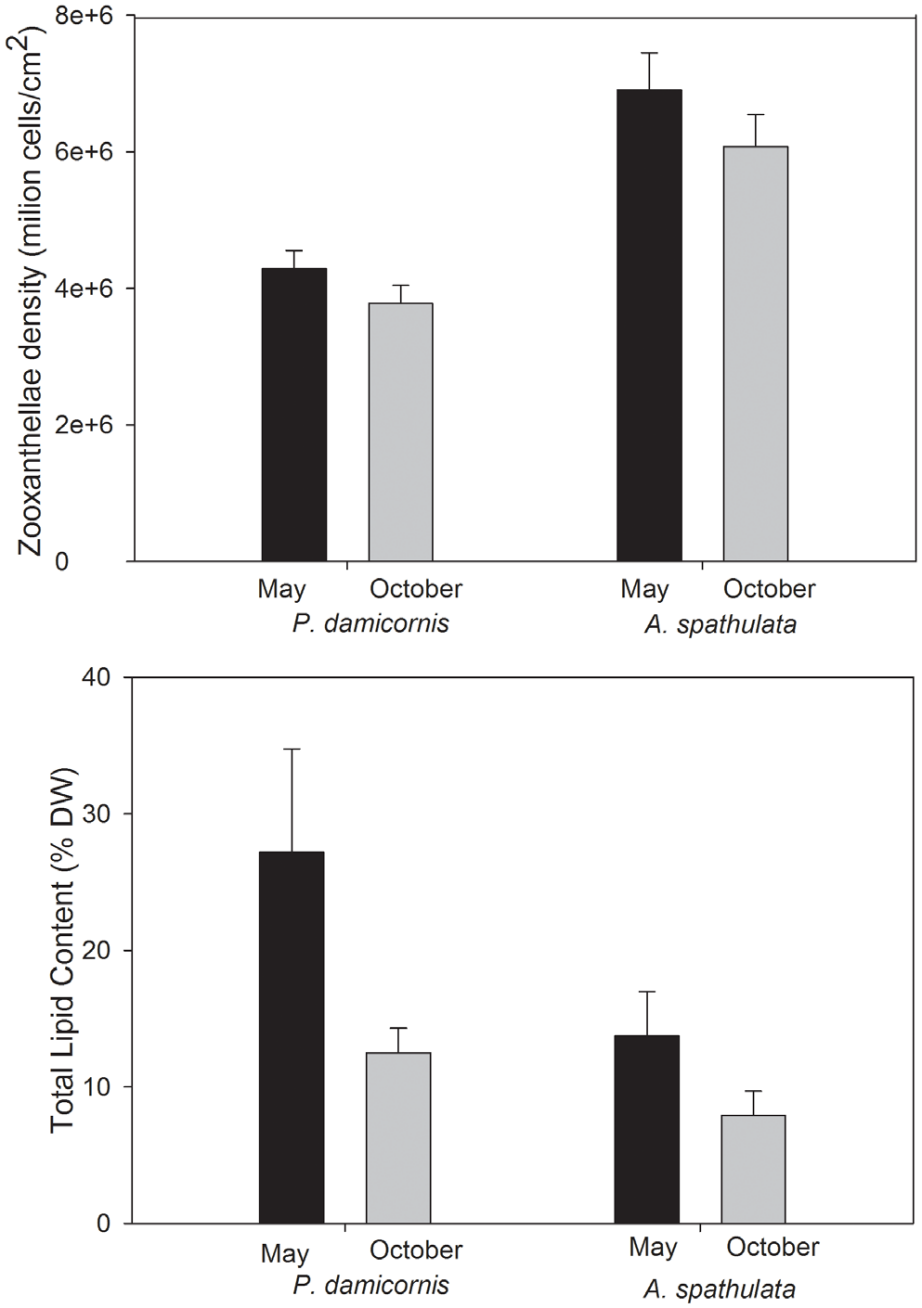
Physiological condition, specifically A) total lipid content and B) zooxanthellae density, in May and October in the two reef-building corals *P. damicornis* and *A. spathulata*.

All the sampled corals survived the entire study period. Colony condition was found to vary between May and October in both coral species (Fig. 2). Specifically, a significant decline in total lipid content was observed in October compared to May (Table 1, 2; Fig. 2). In *A. spathulata* energy reserves in October were almost half compared to May (declined from 13.7 (±7.5) % to 7.8 (±1.8) %), while in *P. damicornis* the decline was two-fold during the same time (Fig. 2). Zooxanthellae density on the other hand, remained constant and did not change significantly between sampling periods in either coral species (Fig. 2). For *P. damicornis*, intra-specific variation in total lipid content was strongly correlated with zooxanthellae density (r = 99, df = 5,42, p<0.001), but no such relationship was found for *A. spathulata*.

**Table 1:** Multiple Regression for zooxanthellae density and total lipid content in *P. damicornis*.

Zooxanthellae	B	StdErr of B	t(42)	p
*Intercept*				
Partial Mortality	−14	834221	−1.7	0.08
Competition	−44	312617	−1.4	0.1
Number of bites	1632	2745	0.5	0.5
Size	174	1044	0.16	0.8
Site	2338	374570	0.06	0.9
Distance from crest	68	61603	1.1	0.2
**Lipid content**	**B**	**StdErr of B**	**t(42)**	**p**
*Intercept*				
Partial Mortality	−23	13.7	−1.7	0.09
Competition	−3.6	5.1	−0.7	0.4
Number of bites	0.008	0.04	0.1	0.8
Size	0.003	0.01	−1.7	0.8
Site	3.96	6.14	0.64	0.5
Distance from crest	−1.6	1.01	−1.5	0.1

**Table 2:** Multiple Regression for zooxanthellae density and total lipid content in *A. spathulata*.

Zooxanthellae	B	StdErr of B	t(18)	p
*Intercept*				
Partial Mortality	−7386077	2909017	−2.5	0.01
Competition	−543263	551519	−0.98	0.3
Number of bites	22837	19454	1.17	0.24
Size	−487	643	−0.75628	0.4
Site	−510264	500279	−1.019	0.3
Distance from crest	−121255	55915	−2.16854	0.03
**Lipid content**	**B**	**StdErr of B**	**t(42)**	**p**
*Intercept*				
Partial Mortality	−2.1	18.5127	−0.11	0.9
Competition	0.7522	3.5098	0.21	0.8
Number of bites	−0.04	0.1238	0.33	0.7
Size	0	0.004	−0.05	0.9
Site	−1.87	3.2105	−0.58	0.5
Distance from crest	0.9	0.3558	2.57	0.01

*P damicornis* showed a high variation within colonies in partial mortality (between 0 and 20%), number of fish bites (between 0 and 392 bites per hour) and total lipid content (between 1.5 and 80% dw). By comparison, intraspecific variation in partial mortality and disturbance rates for *A. spathulata* were much smaller (Fig. 1).

In *P damicornis*, partial mortality, number of fish bites, competition, distance from crest and size were poor predictors of both lipid content (Multiple Regression total lipid content r^2^ = 11, df = 5,42, p = 0.27; Table 1); and zooxanthellae density (r^2^ = 25, df = 5, 42, p = 0.3; Table 1): the regressions explained only a very small proportion of the total variation (<12%). Conversely in *A. spathulata*, partial mortality and distance from crest were found to have a significant effect on both total lipid content and zooxanthellae density (Multiple Regression total lipid content *A. spathulata* r^2^ = 14, df = 5,42, p = 0.2; zooxanthellae density r^2^ = 26, df = 5,42, p = 0.02; Table 2). In particular, total lipid content increased with distance from crest, while zooxanthellae density declined with increasing partial mortality and distance from crest (Table 2).

## Discussion

This is the first study that attempts to relate intraspecific variation in physiological condition of scleractinian corals to small-scale differences in chronic disturbances, such as fish predation. It is well known that coral colonies living in close proximity may exhibit vastly different demographic rates [6], [8], [45], [62], possibly reflective of differences in their disturbance history and subsequent energy allocation [27], [63]. The difficulty in making this link is that very subtle differences in disturbance regimes, operating at any time in the lifetime of each coral, may lead to marked differences in contemporary condition and fitness of individual coral colonies. We acknowledge that the current study provides very limited insights on lifetime differences among closely positioned colonies, mainly due to the limited observational periods, and the range of factors that may be impacting on individual coral colonies. However, it is interesting that we saw no significant temporal shifts in rates of partial mortality, competition and predation between the two observational periods. The high degree of constancy in background mortality may be evidence that there is a high stability in terms of routine mortality.

Under low levels of background mortality, demographic models of scleractinian corals predict constant growth and fecundity of individual colonies, enabling rapid recovery following major acute disturbance [4], [36], [64]. However, in the present study, even within relatively constant rates of biological and physical disturbances, the incidence of injuries still varied among colonies. For instance, some coral colonies did not receive any fish bites in either May or October. Similarly, some colonies of *P. damicornis* that were not injured in May showed partial mortality in October, while some colonies never showed partial mortality. In the long term, these differences among colonies may likely be responsible for important inter-colony differences in condition and fitness. Importantly, variation in the disturbance history of individual colonies may have important ramifications for their long-term fate, especially during major disturbances (e.g., climate-induced coral bleaching).

Not unexpectedly, this study revealed marked intraspecific variation in the physiological condition of both *A. spathulata* and *P. damicornis*. However, these differences were only partially explained by inter-colony differences in rates of partial mortality, competition, predation, colony size or the position of the colony relative to the reef crest. Comparing to other studies, which documented a lipid level of 35% in tissue of *P. damicornis* [56], [65], this study found a lower lipid content (27% dw). Conversely, zooxanthellae density was found to be higher (3.0 cells/cm^2^ in May) than what reported in the literature (3.0 cells/cm^2^, [66]). Also differences in colony condition, specifically in total lipid content, were greater among adjacent colonies of *P. damicornis* than when compared to colonies of *A. spathulata,* revealing intraspecific differences in physiological condition and in susceptibility to chronic disturbances. These differences suggest that coral physiological condition can be more variable than predicted with the outcome depending, in part, on flow, partial mortality, and position of the colony.

Predation rates on coral colonies were higher in October than in May in both coral species, especially in *P. damicornis*. Similarly, coral grazing parrotfishes have been shown to exhibit higher feeding rates in October compared to April on the GBR [67]. For parrotfishes, temporal differences in feeding rates have been previously attributed to differences the nutritional quality of colonies associated with gametogenesis [68]. For butterflyfishes, which tend to take very shallow bites [69], it is unlikely that gametogenesis of the corals would influence feeding behavior, but changes in the nutritional content may still occur within and among coral colonies. For instance mucus production can drive feeding preferences in butterflyfishes [70], [71]. In October, colonies may have released more mucous as a stress response to environmental changes [72] and this discharge may have increased their desirability as food source. The observed differences in bite rates could also be due to seasonal differences in the metabolic rate of food demands of the fishes themselves.

Chronic disturbances were found to affect physiological condition only in. *A. spathulata*, which exhibited strong intraspecific variation that was explained to a large extent by inter-colony differences in biological disturbances and physical position, however these differences were not observed in *P. damicornis*. Even though both study species (*A. spathulata* and *P. damicornis*) are shallow, fast-growing, branching corals, they have slightly different life-histories strategies, which can explain observed differences. *P. damicornis* is a brooding, opportunistic coral which colonizes very disturbed habitats and it is one of the most resilient corals [46]. These characters may explain why *P. damicornis* was more resilient to chronic background disturbances than *A. spathulata*, which instead seems to dominate communities in relatively stable environments [46]. *A. spathulata* showed higher lipid reserves with increasing distance from the crest and lower symbiont density with increasing partial mortality and distance from crest. The observed increase in total lipid content with distance from crest may be due to the higher energetic cost of this reef habitat.

The reef crest is a shallow wave-exposed habitat, where water flow strongly influences organisms mechanically and physiologically with important consequences on community structure [73]. To avoid hydrodynamic dislodgment, colonies on the crest may need to invest more resources in growth to reach the dislodgment threshold [73], but since energy is limited within a colony, if more resources are allocated to increase colony size, less energy will be available to store. The findings from this study suggest that colonies in intermediate position between reef crest and reef flat have a better performance than conspecific on the crest. However, even though the reef crest is an energetic costly habitat, the high flow can positively affect colonies as they can benefit from it for feeding and escretion [74]. Together with light, flow is a critical abiotic factor affecting colony condition [74]. Colonies exposed to high flow generally have higher skeletal density, higher protein concentration, zooxanthellae density, chlorophyll content, and higher number and size of oocytes compared to colonies exposed to lower flow conditions [74]. Flow enhances zooxanthellae density and photosynthesis due to the enhanced nutrient supply [75], and can explain the decreasing zooxanthellae density with distance from crest found in this study.

The symbiotic relationship between the zooxanthellae and the host may be affected by a variety of internal and external factors and processes, the composition of which still has not been fully investigated [54], [76]. Findings from this study suggest that increasing partial mortality and distance from crest may lead to a decline in density of *Symbiodinium*. Not many studies have shown differences in zooxanthellae density among reef habitats regardless of depth. For instance Strickland [76] did not find any difference in zooxanthellae density with increasing distance from the reef crest or location along the reef. Conversely, zooxanthellae within the same reef habitat have been shown to vary with environmental fluctuations and season cycles [54], [77], [78].

Despite consistency in levels of routine or background mortality, the lipid content within coral tissues consistently declined across all coral colonies between May and October in both *P. damicornis* and *A. spathulata*. The decline in total lipid content observed in October in both coral species may partly be explained by sustained and ongoing rates of background mortality, though the declines in may also reflect limited productivity during winter months, due to both reduced temperature and reduced day length [79]. Zooxanthellae supply corals with an excess of lipids and a limitation in their activity can results in a decline in lipid reserves [56], [80]. Stimson [56] documented a decrease in total lipid content following about one month in *P. damicornis* due to light limitation. Corals tend to consume their lipid reserves when maintenance costs of a colony exceed carbon acquisition [22], during environmental unfavourable conditions such as limited light [6], [58], [81], during reproductive events [82]–[84] or whenever an increase in energy demand occurs such as the development of a tumor in coral tissue [85].

Large colonies generally have greater regenerative abilities [6], [8], greater growth [31], are more fecund [17] and have lower rates of total mortality compared to smaller colonies [6], [15]. Consequently we were expecting larger colonies to be more resilient to chronic background disturbances than smaller ones. Conversely, in the present study chronic disturbances had a similar effect on physiological condition of colonies regardless of the size, suggesting that larger colonies are not necessary more resilient than smaller colonies. Similar incidence of chronic disturbances on coral colonies regardless of the size also suggests a lack of size-specific susceptibility to agents of coral mortality [86]. Other studies documented a lack of differences in resilience between small and large colonies [87], [88]. For instance *S. siderea* exposed to partial mortality continued to dedicate resources to reproduction even after the colony had shrunk below their size of maturation while larger colonies reduced their fecundity [88]. Often recent injuries play a bigger role than size in predicting colony fate [89]. Large colonies with higher partial mortality may die before small colonies with no injuries [89].

Extensive research effort has focused on understanding the ability of reef corals to withstand and absorb disturbances, thereby contributing to the persistence and resilience of coral colonies, populations and species [1], [3], [12], [42], [49], [90], [91]. Quantifying the effects of essentially routine and ongoing disturbances on colony condition and assess intraspecific differences in colony condition added to this understanding and it is critical because background mortality influences recovery capacity, time and vulnerability to future disturbances.

This study documented significant effects of partial mortality and distance from crest on zooxanthellae density in *A. spathulata* with important ecological consequences for recovery capacity in the aftermath of climate change. A reduction in performances arising from these sub-lethal stressors, is likely to reduce colony resilience and hence increase chances of whole-colony mortality so that colonies suffering from partial mortality may not survive a subsequent acute disturbance. The approach used here, investigating drivers of colony-condition and their energetic consequences for colony resilience, provides a strong framework for predicting resistance, recovery capacity and resilience of reef-building corals. If colonies in poor physiological conditions (e.g. less resilient) are more susceptible to bleaching, disease and other stressors, colonies capable of maintaining a higher physiological condition may have a distinct ecological advantage [22], [92]. Consequently, colonies of *A. spathulata*, with high partial mortality rates and located on the reef crest, may have a lower potential to withstand and recover from environmental changes compared to conspecific with lower rates of partial mortality and located in intermediate habitats. The observed differences in physiological conditions could have a strong bearing on the selectivity of major disturbances and the capacity of corals to withstand major disturbances, and thereby adapt to changing conditions.

This study is the first to document significant intra-specific variation in background mortality and colony condition, the next step is to investigate whether this variation impacts individual vulnerability of corals. If so, this will provide strong incentive to reduce background levels of stresses (e.g. control all the factors that routinely injure colonies such as predation or anchoring) as a sure way to increase resilience of corals subject to inevitable increases in acute disturbances in association with global climate change.
